# Fibrosis-Associated Signaling Molecules Are Differentially Expressed in Palmar Connective Tissues of Patients with Carpal Tunnel Syndrome and Dupuytren’s Disease

**DOI:** 10.3390/biomedicines10123214

**Published:** 2022-12-11

**Authors:** Ivo Tripković, Marin Ogorevc, Dubravka Vuković, Mirna Saraga-Babić, Snježana Mardešić

**Affiliations:** 1 Department of Plastic Surgery, University Hospital Split, 21000 Split, Croatia; 2 Department of Anatomy, Histology and Embryology, University of Split School of Medicine, 21000 Split, Croatia; 3 Department of Dermatovenerology, University Hospital Split, 21000 Split, Croatia

**Keywords:** carpal tunnel syndrome, dupuytren contracture, FGFR1, FGFR2, CTGF, TGF-beta, Syndecan-1

## Abstract

Carpal tunnel syndrome (CTS) and Dupuytren’s disease (DD) are fibrotic conditions that affect the connective tissue of the hand and limit its functionality. The exact molecular mechanism underlying the fibrosis is unknown, and only some profibrotic factors have been investigated. In this cross-sectional study, we analyzed the expression of FGF signaling pathway molecules associated with fibrotic changes in the palmar fascia and the flexor retinaculum of 15 CTS patients and both clinically affected and unaffected palmar fascia of 15 DD patients, using immunofluorescence techniques. The expression of FGFR1, FGFR2, and CTGF in the blood vessel walls and surrounding connective tissue cells differed significantly between the analyzed groups, with changes in expression present even in clinically unremarkable tissues from DD patients. We also found altered expression of the analyzed factors, as well as TGF-β1 and syndecan-1 in DD-associated sweat glands, possibly implicating their role in the pathophysiology of the disease. The increased expression of profibrotic factors in the clinically unaffected palmar fascia of DD patients may indicate that more extensive excision is needed during surgical treatment, while the profibrotic factors could be potential targets for developing pharmacological therapeutic strategies against DD-associated fibrosis.

## 1. Introduction

Carpal tunnel syndrome (CTS) is the most common compression neuropathy. It is defined as a compression of the median nerve at the level of the wrist joint associated with decreased function of the nerve at that level [1]. Idiopathic CTS has no known cause, unlike secondary CTS which is associated with diseases, such as diabetes mellitus, autoimmune diseases (rheumatoid arthritis, scleroderma etc.), and hypothyroidism [2]. It can be either acute or chronic, the latter being much more common [3]. Based on clinical examinations and nerve conduction studies, it has been approximated that one in every five subjects who complain of symptoms, such as pain, numbness, and a tingling sensation in the hands, could have CTS [4]. The connective tissue fasciae surrounding the median nerve have been implicated in the pathogenesis of CTS [5]; however, considering that the pathophysiology of idiopathic CTS is not fully clear, definitive treatment strategies have not been established. Treatment should be selected considering various factors, such as the stage of the disease, the severity of the symptoms, or the patient’s preference [6]. In recent literature, surgical treatment has been reported to be more effective than splinting and other conservative treatments [7,8].

Dupuytren’s disease (DD) is a common fibroproliferative disorder of the hand that is often progressive and eventually can cause contractures of the affected fingers [9]. It is a multifactorial and complex disease and has been reported to be associated with inherited genetic markers, alcohol and tobacco use, and different systemic diseases such as diabetes and epilepsy [10,11,12]. One of the main factors involved in the development of this disease is the proliferation of myofibroblasts in the affected tissues. Myofibroblasts share characteristics of both fibroblasts and smooth muscle cells, and they may be responsible for the tissue contracture found in the initial phases of DD [13,14]. The treatment of DD is complex, and it involves surgical and non-surgical approaches, all of them with a unique goal of eliminating the affected tissue. However, the most effective treatments are the surgical removal of the fibrous cords causing the patient’s symptoms by fasciectomy or fasciotomy [15,16]. In most cases, the evolution of DD is progressive and irreversible, and the risk of relapse after surgical excision is high. The risk of disease recurrence ranges between 8% and 71% [11], making additional research on the causes and factors related to this disease necessary.

The etiology of CTS and DD is believed to be associated with changes of blood vessel walls and sweat glands, as well as numerous profibrotic factors, including fibroblast and connective tissue growth factors (FGF, CTGF), transforming growth factors (TGF-β) and transmembrane proteoglycans [17,18]. Blood vessels generally have three layers: an intima consisting of endothelium and subendothelial connective tissue, a media with multiple smooth muscle cell layers, and an adventitia of connective tissue. The smallest blood vessels, capillaries, consist of endothelium surrounded by pericytes [19]. Eccrine sweat glands consist of a deeper, coiled region containing the entire secretory portion and the beginning of the duct, and a more superficial, straight region containing the rest of the duct. The secretory portion is made up of luminal and myoepithelial cells, while the duct is composed of luminal and basal cells [20].

Fibroblast growth factors (FGFs) are a group of signaling molecules that contribute to the growth, differentiation, survival, morphogenesis, angiogenesis, and repair of a variety of tissue types [21]. Of the 22 FGFs that are currently known, 18 bind to and activate a family of receptor tyrosine kinases known as FGF receptors (FGFRs) [22]. While there are only four FGFRs (FGFR1–4), all of them, except FGFR4, have additional isoforms that display specific expression patterns. Some are exclusively expressed in epithelial cells, while others only in mesenchymal cells [23]. Activation of FGFRs on endothelial cells promotes angiogenesis, mainly through FGFR1-mediated proliferation, with a contribution to cell motility from FGFR2 activation [24]. Vascular smooth muscle cells (VSMCs) primarily express FGFR1, and activation of FGF-signaling induces the conversion of VSMC from a contractile to a proliferative phenotype [25]. By now, FGFR2 expression has been described in luminal cells of sweat gland secretory portions [26]; however, FGFR1 expression in sweat glands has not been described in any study to the best of the authors’ knowledge.

Transforming growth factor β (TGF-β) and connective tissue growth factor (CTGF) are two of the most studied profibrotic factors. Three homologs of TGF-β exist, with TGF-β1 being the main representative of the entire superfamily. It is associated with multiple processes, such as proliferation, apoptosis, differentiation, and wound healing [27]. TGF-β1 has been shown to promote tissue fibrosis by inducing synthesis and inhibiting the degradation of extracellular matrix (ECM) components [28], and it has a role in angiogenesis and maintaining vascular wall integrity [29]. Both secretory portions and eccrine sweat gland ducts have been shown to express TGF-β1 [30]. CTGF is an ECM protein involved in differentiation, proliferation, adhesion, and angiogenesis. It regulates intercellular signaling by binding to cell surface receptors, cytokines, and ECM components. It also acts as a mediator of ECM turnover, both in physiologic conditions and tissue fibrosis [31]. Interestingly, the expression of CTGF is regulated by TGF-β, with sustained CTGF expression demonstrated even after transitory TGF-β stimulation [32]. CTGF expression has been described both in endothelium and VSMCs, with it acting as a mediator of their interactions during vascular remodeling [33].

Syndecans are transmembrane proteoglycans that can regulate cell behavior. Four types are found in mammals, with syndecan-1, also known as CD138, being the founding member. Syndecan-1 is primarily expressed by epithelial cells, but some stromal cells also show its expression. It can bind a variety of ligands, such as growth factors, chemokines, morphogens, and ECM components. It can also associate with other transmembrane receptors, such as FGFRs, and contributes to the internalization of ligand–receptor complexes [34].

Taking all of this into consideration, we hypothesize that cell proliferation and profibrotic factors will be increased in the palmar fascia of DD patients, especially in areas with marked fibrotic changes. The objective of this study is to analyze the proliferation and expression of FGFR1, FGFR2, and CTGF in the cells of blood vessel walls and surrounding connective tissue of CTS and DD patients. Furthermore, TGF-β1 and syndecan-1 expression, in addition to the previously mentioned factors, were analyzed in the eccrine sweat glands of DD patients. Our findings may determine whether the flexor retinaculum is involved in the pathophysiology of CTS, contribute to the understanding of the molecular mechanisms involved in fibrosis associated with DD, and potentially reveal novel therapeutic strategies for its management.

## 2. Materials and Methods

### 2.1. Tissue Acquisition and Processing

Palmar fascia and flexor retinaculum samples were obtained from routine surgical procedures at the Department of Plastic Surgery of the University Hospital in Split in the period between March 2021 and March 2022. All patients gave informed consent for their tissues to be used in the study, and all performed procedures were approved by the Ethical and Drug committee of the University Hospital in Split (class: 500-03/21-01/36, registry number: 2181-147-01/06/M.S.-20-02; accessed on 26 February 2021) and in accordance with the Declaration of Helsinki and other relevant national guidelines and regulations. Samples were obtained from a total of 15 patients with CTS and 15 patients with DD and were separated into four groups: palmar fascia from CTS patients (control samples), flexor retinaculum from CTS patients, fibrotic cords from patients with DD, and clinically unaffected palmar fascia adjacent to fibrotic cords from patients with DD. Each group contained 15 samples, which was a sufficient sample size as calculated by Mead’s resource equation. All 15 patients with DD had grade 2 DD, as described by Townley et al. [35]. Samples were processed as previously described [36]. Briefly, all samples underwent identical initial processing steps: fixation in a 4% paraformaldehyde solution in phosphate-buffered saline (PBS), dehydration in ethanol solutions of increasing concentration (up to 100% ethanol), clearing with toluene solutions, infiltration with melted paraffin in an oven, embedding by cooling at room temperature, trimming excess paraffin, cutting 5 µm-thick serial sections using a microtome, and mounting the sections on glass slides. Afterward, the tissues were deparaffinized in xylene and rehydrated in graded water–ethanol solutions to prepare them for staining with hematoxylin and eosin, Mallory trichrome stain, or immunofluorescent stain.

### 2.2. Hematoxylin and Eosin Staining

The samples were immersed in a hemalaun solution for 10 min and subsequently washed with distilled water, followed by fixation in hot water. An eosin solution was applied for 10 min and washed with distilled water. The samples were dehydrated again using graded ethanol solutions and underwent three rounds of treatment in xylene solutions. Finally, the samples were coverslipped using Canada balsam.

### 2.3. Mallory Trichrome Staining

The tissue samples underwent postfixation with Bouin’s fixative in a water bath at 60 °C for 1 hr. Afterward, acid fuchsin was applied for 3 to 5 min, then phosphomolybdic acid for 5 min, followed by a combination of aniline blue and orange G stains for 10 min. Graded alcohol and xylene solutions were used for dehydration prior to coverslipping the samples with Canada balsam.

### 2.4. Immunofluorescent Staining

Antigen retrieval was performed by heating samples at 95 °C for 30 min in sodium citrate buffer (pH 6.0). The samples were then allowed to cool to room temperature and rinsed in PBS. To prevent non-specific staining, protein blocking buffer (Protein Block ab64226, Abcam, Cambridge, UK) was applied for 20 min in a humid chamber, followed by incubation with a combination of primary antibodies (Table 1) overnight in the humid chamber. The samples were subsequently rinsed in PBS twice, and incubation with appropriate secondary antibodies (Table 1) was performed for an hour in the humid chamber. Next, samples underwent three rounds of rinsing in PBS and DAPI (4′,6-diamidino-2-phenylindole) was applied for 2 min to counterstain nuclei. After washing the sections with distilled water, the slides were air-dried and coverslipped with a mounting medium (ImmuMount, Thermo Shandon, Pittsburgh, PA, USA). Omitting primary antibodies from the protocol resulted in the absence of specific staining and was used as a control for the specificity of staining. The samples were examined with a fluorescent microscope (Olympus BX61, Olympus Corporation, Tokyo, Japan), and micrographs were captured using a mounted digital camera (Nikon Ri-D2, Nikon Corporation, Tokyo, Japan).

### 2.5. Immunofluorescence Signal Quantification

We used individual cell counts to quantify the immunohistochemical expression (i.e., spatial expression) of Ki-67, syndecan-1, FGFR1, FGFR2, CTGF, and TGF-β in the blood vessels, connective tissue, and sweat glands of our samples. After morphological analysis to determine tissue quality, the 10 most representative samples per analyzed group were used for quantification. For each analyzed protein, we merged the specific protein stain image with its respective nuclear DAPI stain image using the layering option in Adobe Photoshop (Adobe, San Jose, CA, USA). The merged images were opened in ImageJ software (NIH, Bethesda, MD, USA), and 100 cells of a certain cell type were counted and marked using the Multipoint tool for each analyzed sample. Then, we counted how many of the 100 marked cells displayed positive staining for the analyzed protein and calculated the relative percentage of positive cells, which was used for further statistical analysis. Only nuclear staining was considered positive for Ki-67, while membranous and/or cytoplasmatic staining was considered positive for the other analyzed proteins. We counted exactly 100 cells per cell type in each sample to reduce the influence of the variability and heterogeneity of our samples on our results.

### 2.6. Statistical Analysis

All the results are displayed as the mean ± standard deviation of the relative percentage of positive cells. The normality of the distribution of data was tested using the D’Agostino–Pearson normality test. The statistical significance of the difference in Ki-67 and syndecan-1 expression was determined using one-way ANOVA with Uncorrected Fisher’s LSD post hoc test. For FGFR1, FGFR2, CTGF, and TGF-β1, the significance of differences of expression in blood vessels and connective tissue cells was determined by Welch ANOVA and Dunnett’s T3 post hoc test, while ordinary one-way ANOVA and Tukey’s post hoc test were used for expression in sweat glands. Statistical significance was set at *p* < 0.05. Effect size was measured by Cohen’s d value, and d > 0.8 was considered a large effect size.

## 3. Results

The term ‘expression’ used in our descriptive findings is a synonym for the intensity of immunofluorescent staining of the analyzed structure, while ‘increased/decreased expression’ in the description of our statistical analysis represents a larger/smaller relative percentage of positive cells for the analyzed factor.

### 3.1. Haematoxylin and Eosin Staining (H&E)

**Figure 1:**
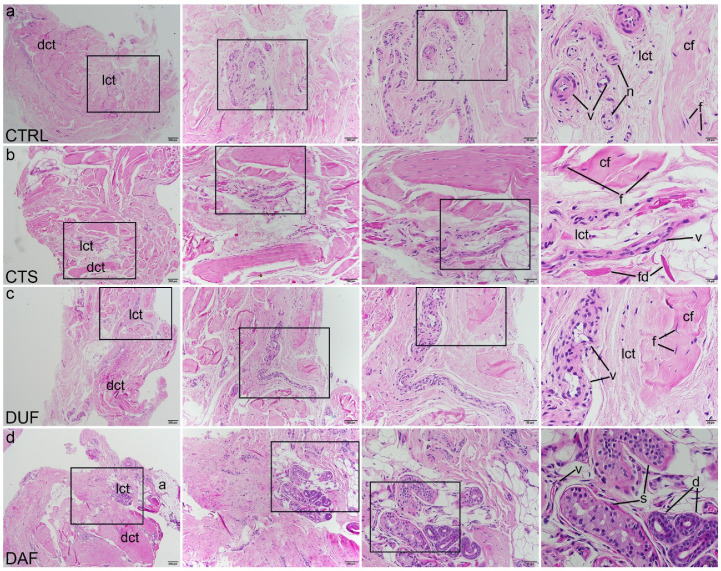
Haematoxylin and eosin staining of connective tissues of CTS and DD patients. Rectangles mark the area presented at a higher magnification in the following image. Control samples of healthy palmar fascia (CTRL) consist of dense connective tissue (dct) with bundles of collagen fibers (cf) with fibroblasts (f) and areas of loose connective tissue (lct) where blood vessels (v) and nerves (n) can be seen (**a**); in the flexor retinaculum of CTS patients, dense connective tissue (dct) has more densely packed collagen fibers (cf) and is separated by loose connective tissue (lct) in which blood vessels (v) and fibrinogen deposits (fd) can be observed (**b**); clinically unaffected palmar fascia of DD patients (DUF) displays areas of dense connective tissue (dct) with thick strands of collagen fibers (cf), separated by areas of loose connective tissue (lct) containing blood vessels (v) (**c**); samples of affected palmar fascia of DD patients (DAF) show large areas of dense connective tissue (dct) containing dense collagen fibers (cf) with many fibroblasts (f), separated by loose connective tissue (lct) containing blood vessels (v) and adipocytes (a); at places, ducts (d) and secretory portions (s) of sweat glands are seen (**d**); 1st column, 40× magnification, scale bar 200 µm; 2nd column, 100× magnification, scale bar 100 µm; 3rd column, 200× magnification, scale bar 50 µm; 4th column, 400× magnification, scale bar 20 µm.

Haematoxylin and eosin (H&E) staining revealed healthy palmar fascia (control sample) was predominantly built of irregular dense connective tissue, while areas of loose connective tissue contained blood vessels and small nerves (Figure 1a). In contrast, the flexor retinaculum of CTS patients showed more densely packed bundles of collagen fibres (at places showing parallel orientation), while areas of blood vessels and fibrinogen deposits characterized loose connective tissue islands (Figure 1b). In clinically unaffected palmar fascia of DD patients (DUF) dense connective tissue predominated, with areas of loose connective tissue observed around blood vessels (Figure 1c). Cords of palmar fascia affected by DD (DAF) showed highly cellular, densely packed connective tissue, surrounding islands of loose connective tissue with blood vessels and adipocytes, and secretory parts and ducts of eccrine sweat glands (Figure 1d).

### 3.2. Mallory Trichrome Staining (MTC)

**Figure 2:**
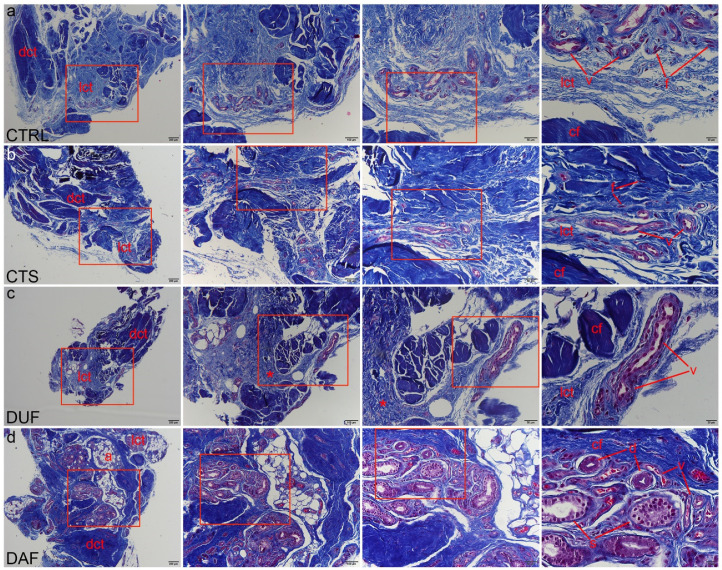
Mallory trichrome staining of connective tissues of CTS and DD patients. Rectangles mark the area presented at a higher magnification in the following image. Healthy palmar fascia samples show dark blue staining of collagen fibers (cf) in dense connective tissue (dct), with lighter blue areas of loose connective tissue (lct) containing blood vessels (v) and fibroblasts (f) (**a**); the flexor retinaculum of CTS patients contains more areas of darker staining dense connective tissue (dct) with collagen fiber bundles (cf), while in areas of loose connective tissue (lct), blood vessels (v) are observed (**b**); the clinically unaffected palmar fascia of DD patients (DUF) also contains areas of dense connective tissue (dct) made up of mostly collagen fibers (cf) and areas of loose connective tissue (lct) with blood vessels (v). Some regions of loose connective tissue display darker blue staining (asterisks), signifying denser collagen packing (**c**); in samples of affected palmar fascia of DD patients (DAF), dark blue staining of collagen fibers (cf) characterizes areas of dense (dct) and loose connective tissue (lct), also surrounding blood vessels (v), adipocytes (a), and secretory parts (s) and ducts (d) of sweat glands (**d**); 1st column, 40× magnification, scale bar 200 µm; 2nd column, 100× magnification, scale bar 100 µm; 3rd column, 200× magnification, scale bar 50 µm; 4th column, 400× magnification, scale bar 20 µm.

Mallory trichrome staining was used to delineate areas of dense connective tissue with dark blue staining of type I collagen fibres, while areas of irregular loose connective tissue showed lighter blue staining. Contents of the control tissue corresponded to those described by the H&E method (Figure 2a). Compared to the control, MTC staining revealed areas of more densely packed collagen fibres in the flexor retinaculum, even in areas around blood vessels (Figure 2b). DUF samples generally contained more dense, dark blue stained connective tissue, which was observed in areas of both dense and loose connective tissue with blood vessels (Figure 2c). Compared to DUF, in DAF samples some blood vessels and components of sweat glands were encircled by the fibrotic cords, composed of densely packed collagen fibres, contrasting the blood vessels inside the less densely packed islands of irregular loose connective tissue (Figure 2d).

### 3.3. Blood Vessel and Connective Tissue Cell Proliferation–Ki-67 and α-SMA Co-Expression

**Figure 3:**
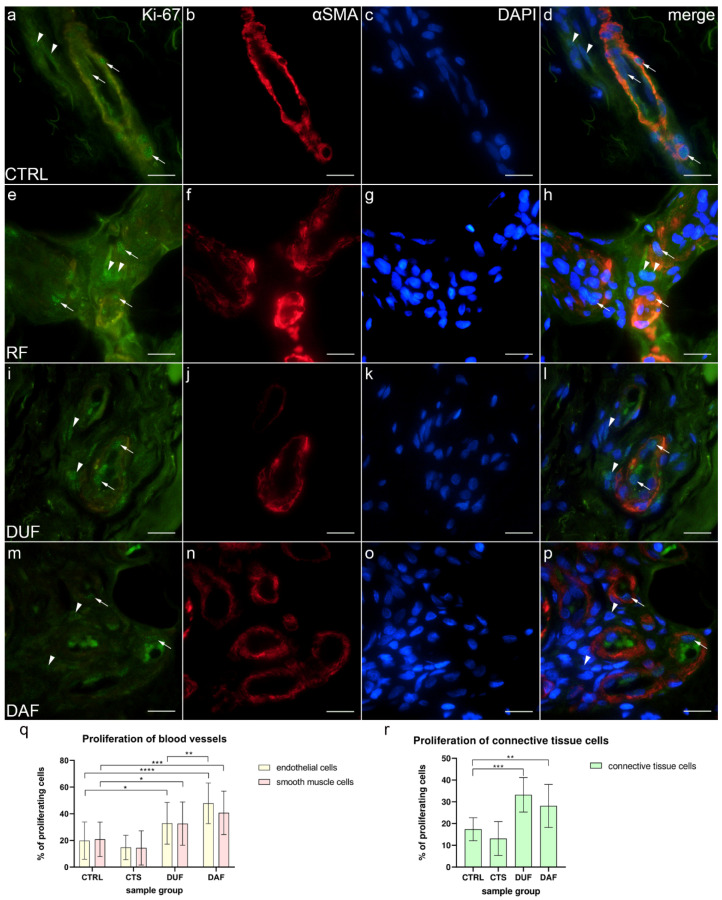
Co-expression of Ki-67 and α-SMA in connective tissues of patients with CTS and DD. CTRL–palmar fascia of CTS patients (**a**–**d**); RF–flexor retinaculum of CTS patients (**e**–**h**); DUF–clinically unaffected palmar fascia of DD patients (**i**–**l**); DAF–clinically affected palmar fascia of DD patients (**m**–**p**). Nuclear Ki-67 expression (**a**,**d**,**e**,**h**,**i**,**l**,**m**,**p**) is seen in blood vessels (arrows) and surrounding connective tissue cells (arrowheads); α-SMA staining shows sections through blood vessels (**b**,**f**,**j**,**n**). DAPI staining displays all cell nuclei (**c**,**g**,**k**,**o**). Double immunofluorescence staining to Ki-67, α-SMA, and DAPI, 1000× magnification, scale bar 50 µm. Statistically significant differences in proliferation rate are displayed by graphs for both blood vessels (**q**) and connective tissue cells (**r**). Error bars show standard deviation; * *p* < 0.05, ** *p* < 0.01, *** *p* < 0.001, **** *p* < 0.001.

The proliferation marker Ki-67 was observed as green granular nuclear staining inside the walls of blood vessels and in the surrounding connective tissue of all analyzed specimens (Figure 3a,e,i,m). Alpha smooth muscle actin (α-SMA) demonstrated smooth muscle cells of the blood vessel wall (Figure 3b,f,j,n), while DAPI staining showed localization of all nuclei (Figure 3c,g,k,o). Merging of the images revealed cells with nuclear Ki-67 staining, i.e., proliferating cells (Figure 3d,h,l,p).

In control samples (CTRL), co-localization of Ki-67 (Figure 3a), α-SMA (Figure 3b), and DAPI staining (Figure 3c) was observed in endothelial and smooth muscle cells of blood vessel walls and occasionally in the surrounding connective tissue cells (Figure 3d). Similarly, in CTS patients, Ki-67 staining (Figure 3e) was co-localized with α-SMA staining (Figure 3f) and DAPI staining (Figure 3g) in the endothelial and smooth muscle cells of blood vessels and in the connective tissue cells (Figure 3h). In DUF samples, an increased number of cells co-expressing Ki-67 (Figure 3i), α-SMA staining (Figure 3j), and DAPI nuclear stain (Figure 3k) was observed in the endothelium of blood vessels and in several connective tissue cells (Figure 3l). Compared to DUF, in DAF samples, co-localizations of proliferating Ki-67-positive cells (Figure 3m), α-SMA (Figure 3n), and nuclear DAPI (Figure 3o) staining was visible in endothelial and smooth muscle cells of blood vessels. Some connective tissue cells also showed nuclear Ki-67 staining (Figure 3p).

Endothelial cells of control samples showed a significantly smaller proliferation rate (19.85% ± 14.01%) compared to endothelial cells of both DUF (32.82% ± 15.65%; *p* = 0.013; d = 0.873) and DAF samples (47.80% ± 15.21%; *p* < 0.0001; d = 1.911). Endothelial cells of DAF samples had a significantly higher proliferation rate than DUF samples (*p* = 0.003; d = 0.971). Vascular smooth muscle cells of control samples also showed a significantly smaller proliferation rate (20.85% ± 12.93%) compared to vascular smooth muscle cells of both DUF (32.59% ± 16.24%; *p* = 0.035; d = 0.800) and DAF samples (40.73% ± 16.29%; *p* = 0.001; d = 1.352). There were no significant differences between control and CTS samples (Figure 3q). Additionally, the proliferation rate of connective tissue cells of control samples (17.41% ± 5.32%) was significantly lower compared to DUF (33.25% ± 7.96%; *p* = 0.001; d = 2.349) and DAF (28.17% ± 9.95%; *p* = 0.009; d = 1.348) samples. There was no difference between control and CTS samples (Figure 3r).

### 3.4. Co-Expression of FGFR1 and α-SMA in Blood Vessels and Surrounding Connective Tissue

**Figure 4:**
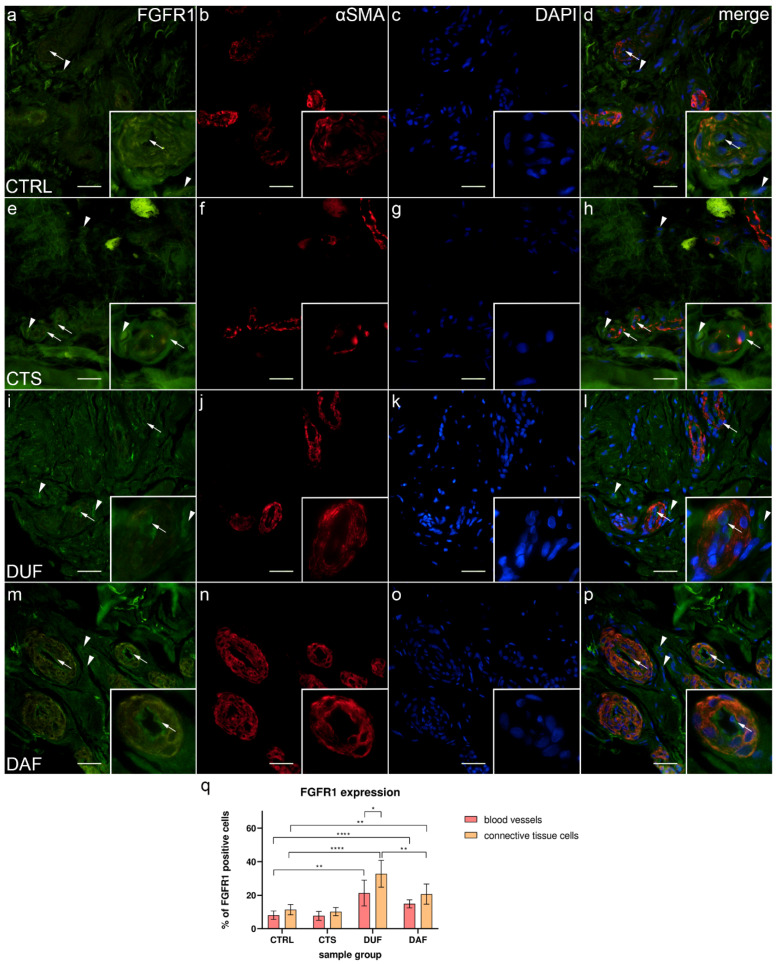
Co-expression of FGFR1 and α-SMA in connective tissues of patients with CTS and DD. CTRL–palmar fascia of CTS patients (**a**–**d**); RF–flexor retinaculum of CTS patients (**e**–**h**); DUF–clinically unaffected palmar fascia of DD patients (**i**–**l**); DAF–clinically affected palmar fascia of DD patients (**m**–**p**). FGFR1 expression (**a**,**d**,**e**,**h**,**i**,**l**,**m**,**p**) is seen in blood vessels (arrows) and surrounding connective tissue cells (arrowheads); α-SMA staining shows sections through blood vessels (**b**,**f**,**j**,**n**). DAPI staining displays all cell nuclei (**c**,**g**,**k**,**o**). Insets (**a**–**p**) reveal the distribution of FGFR1 staining in blood vessels. Double immunofluorescence staining to FGFR1, α-SMA, and DAPI, 400× magnification, scale bar 100 µm. The graph displays statistically significant differences in FGFR1 expression of blood vessel and connective tissue cells (**q**). Error bars show standard deviation; * *p* < 0.05, ** *p* < 0.01, **** *p* < 0.001.

In control samples, weak FGFR1 expression could be seen in endothelial and smooth muscle cells of control sample blood vessel walls, while it was missing in most connective tissue cells (Figure 4a); α-SMA staining characterized smooth muscle cells in the multiple cross sections through blood vessel walls (Figure 4b), while DAPI staining showed nuclei of all structures (Figure 4c,g,k,o). Merging of the images demonstrated only a few vascular smooth muscle cells and connective tissue cells showing moderate FGFR1 expression (Figure 4d). In contrast, CTS patients’ tissue displayed weak FGFR1 expression in the walls of blood vessels, while strong expression was seen in some cells and within the fibrinogen deposits of the connective tissue (Figure 4e). Blood vessels were demonstrated by intense α-SMA staining (Figure 4f), while cell nuclei were DAPI positive (Figure 4g). The merged image showed the strongest FGFR1/ α-SMA co-expression in the wall of blood vessels (Figure 4h). In DUF samples, the strongest expression was seen in some endothelial cells, while weak expression characterized the vascular smooth muscle cells (Figure 4i); α-SMA staining characterized vascular smooth muscle (Figure 4j), while nuclei showed DAPI staining (Figure 4k). Merging of the images revealed the absence of overlapping FGFR1 and α-SMA expression in the muscular vessel wall (Figure 4l). In comparison to DUF, DAF samples had blood vessels with moderate to strong FGFR1 expression in most of the endothelial cells and weak expression in the muscular walls, while areas of moderate expression were observed in the surrounding connective tissue (Figure 4m). In the blood vessels, α-SMA stained vascular smooth muscle cells (Figure 4n), while DAPI stained nuclei (Figure 4o) Overlapping of the images demonstrated that the strongest FGFR1 expression was in the endothelial cells, while smooth muscle cells and connective tissue cells showed weaker expression (Figure 4p).

The expression of FGFR1 in blood vessels of control samples (8.10% ± 2.51%) was significantly lower compared to DUF (21.30% ± 7.70%; *p* = 0.002; d = 2.304) and DAF samples (14.90% ± 2.42%; *p* < 0.0001; d = 2.754). Connective tissue cell FGFR1 expression was also significantly lower in control samples (11.40% ± 3.10%) compared to both DUF (32.80% ± 7.96%; *p* < 0.0001; d = 3.543) and DAF samples (20.70% ± 6.06%; *p* = 0.005; d = 1.932). The expression of FGFR1 in connective tissue cells of DUF samples was significantly higher than in DAF sample connective tissue cells (*p* = 0.008; d = 1.710) and DUF sample blood vessels (*p* = 0.016; d = 1.468). There was no significant difference between control and CTS samples (Figure 4q).

### 3.5. Co-Expression of FGFR2 and α-SMA in Blood Vessels and Surrounding Connective Tissue

**Figure 5:**
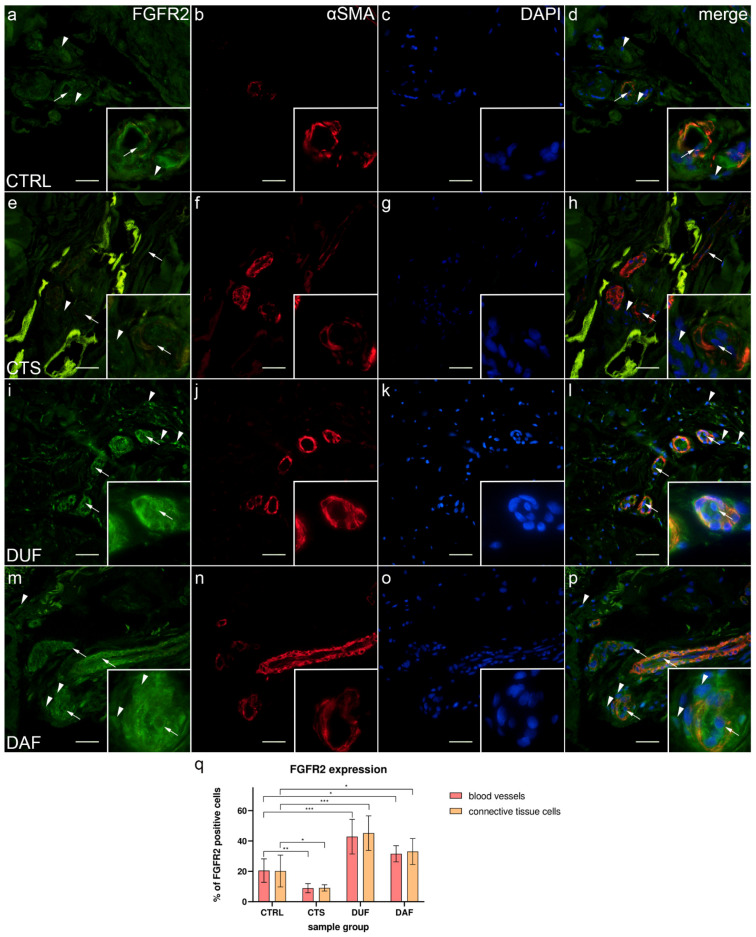
Co-expression of FGFR2 and α-SMA in connective tissues of patients with CTS and DD. CTRL–palmar fascia of CTS patients (**a**–**d**); RF–flexor retinaculum of CTS patients (**e**–**h**); DUF–clinically unaffected palmar fascia of DD patients (**i**–**l**); DAF–clinically affected palmar fascia of DD patients (**m**–**p**). FGFR2 expression (**a**,**d**,**e**,**h**,**i**,**l**,**m**,**p**) is seen in blood vessels (arrows) and surrounding connective tissue cells (arrowheads); α-SMA staining shows sections through blood vessels (**b**,**f**,**j**,**n**). DAPI staining displays all cell nuclei (**c**,**g**,**k**,**o**). Insets (**a**–**p**) reveal the distribution of FGFR2 staining in blood vessels. Double immunofluorescence staining to FGFR2, α-SMA, and DAPI, 400× magnification, scale bar 100 µm. The graph displays statistically significant differences in FGFR2 expression of blood vessel and connective tissue cells (**q**). Error bars show standard deviation; * *p* < 0.05, ** *p* < 0.01, *** *p* < 0.001.

In control samples, moderate to strong FGFR2 expression was visible in both endothelial and smooth muscle cells of blood vessel walls and in some connective tissue cells (Figure 5a); α-SMA staining demonstrated smooth muscle cells in blood vessel walls (Figure 5b), while DAPI staining showed all nuclei (Figure 5c,g,k,o). Merging of the images revealed co-localization of FGFR2/α-SMA positive cells in the vascular wall (Figure 5d). In comparison, weak to moderate FGFR2 expression was seen in most blood vessel cells of CTS and in the connective tissue cells, which also contained fibrin deposits (Figure 5e). Sections through the blood vessels were demonstrated by α-SMA staining (Figure 5f), while their nuclei by DAPI stain (Figure 5g). The merged image showed FGFR2/α-SMA co-expression in blood vessels, preferably in the smooth muscle cells (Figure 5h). Compared to control samples, expression of FGFR2 was much stronger in both the blood vessel walls of and surrounding connective tissue cells of DUF samples (Figure 5i). Several blood vessels were visualized by strong α-SMA staining (Figure 5j), while DAPI stained cell nuclei (Figure 5k). Merging of the images revealed that the strongest co-expression of FGFR2/α-SMA was in the walls of blood vessels (Figure 5l). In DAF samples, strong FGFR2 expression characterized the vascular wall and some connective tissue cells (Figure 5m); α-SMA staining showed strong expression in blood vessel smooth muscles and endothelium (Figure 5n). Overlapping of the images showed that the strongest FGFR2/α-SMA co-expression was present in the smooth muscle cells of blood vessels (Figure 5p).

The expression of FGFR2 in blood vessels of control samples (20.50% ± 7.74%) was significantly higher compared to CTS samples (8.90% ± 3.00%; *p* = 0.005; d = 1.978), while it was significantly lower than in DUF (42.90% ± 11.41%; *p* = 0.001; d = 2.298) and DAF samples (31.60% ± 5.32%; *p* = 0.010; d = 1.672). Connective tissue cell FGFR2 expression was also significantly higher in control samples (20.20% ± 10.49%) compared to CTS samples (9.10% ± 2.08%; *p* = 0.044; d = 1.468), and it was significantly lower compared to both DUF (45.20% ± 11.40%; *p* = 0.0004; d = 2.282) and DAF samples (33.10% ± 8.57%; *p* = 0.044; d = 1.347). There was no significant difference in FGFR2 expression between DUF and DAF samples (Figure 5q).

### 3.6. Co-Expression of CTGF and α-SMA in Blood Vessels and Surrounding Connective Tissue

**Figure 6:**
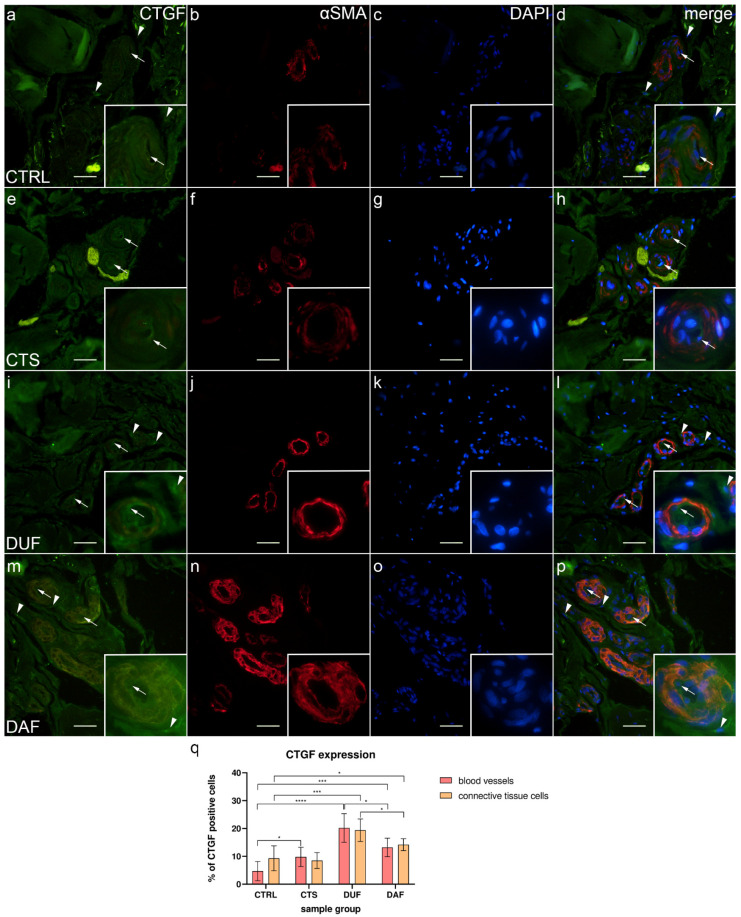
Co-expression of CTGF and α-SMA in connective tissues of patients with CTS and DD. CTRL–palmar fascia of CTS patients (**a**–**d**); RF–flexor retinaculum of CTS patients (**e**–**h**); DUF–clinically unaffected palmar fascia of DD patients (**i**–**l**); DAF–clinically affected palmar fascia of DD patients (**m**–**p**). CTGF expression (**a**,**d**,**e**,**h**,**i**,**l**,**m**,**p**) is seen in blood vessels (arrows) and surrounding connective tissue cells (arrowheads); α-SMA staining shows sections through blood vessels (**b,f,j,n**). DAPI staining displays all cell nuclei (**c**,**g**,**k**,**o**). Insets (**a**–**p**) reveal the distribution of CTGF staining in blood vessels. Double immunofluorescence staining to CTGF, α-SMA, and DAPI, 400× magnification, scale bar 100 µm. The graph displays statistically significant differences in CTGF expression of blood vessel and connective tissue cells (**q**). Error bars show standard deviation; * *p* < 0.05, *** *p* < 0.001, **** *p* < 0.001.

Weak to moderate CTGF expression was present at the inner surface (endothelium) of most blood vessels, while some connective tissue cells and fibers showed strong expression in control samples (Figure 6a); α-SMA staining showed multiple cross sections through blood vessels (Figure 5b), while DAPI staining showed all nuclei (Figure 6c). Merging of the images revealed an absence of CTGF/α-SMA co-expression in the muscular part of blood vessels (Figure 6d). In CTS samples, weak CTGF expression could be seen in the wall of blood vessels and occasionally in some areas of the surrounding connective tissue. The aforementioned deposits were also present, particularly around the thick-walled blood vessels (Figure 6e); α-SMA staining characterized vascular smooth muscle cells (Figure 6f), while DAPI stained cell nuclei (Figure 6f). The merged image shows moderate CTGF/α-SMA co-expression in certain muscle cells of blood vessels (Figure 6h). Compared to CTS, in DUF samples, moderate to strong CTGF expression was seen throughout the walls of blood vessels and in the surrounding connective tissue (Figure 6i). Multiple blood vessel cross sections were visualized by α-SMA staining (Figure 6j). Merging of the images (Figure 6i–k) revealed CTGF/α-SMA co-expression in the smooth muscles of blood vessels (Figure 5l). In DAF samples, weak to moderate CTGF expression was seen throughout the vascular wall, while moderate to strong expression characterized connective tissue cells (Figure 6m); α-SMA staining characterized vascular muscle cells (Figure 6n), while DAPI stained cell nuclei (Figure 6o). The merged image showed CTGF/α-SMA co-expression the vascular smooth muscle cells (Figure 6p).

The expression of CTGF in blood vessels of control samples (4.70% ± 3.47%) was significantly lower compared to CTS (9.80% ± 3.46%; *p* = 0.023; d = 1.473), DUF (20.20% ± 5.16%; *p* < 0.0001; d = 3.526), and DAF samples (13.20% ± 3.36%; *p* = 0.0002; d = 2.490). Connective tissue cell CTGF expression was also significantly lower in control samples (9.30% ± 4.47%) compared to DUF (19.40% ± 4.06%; *p* = 0.0003; d = 2.364) and DAF samples (14.20% ± 2.15%; *p* = 0.044; d = 1.396). CTGF expression was higher in DUF than in DAF samples, both in blood vessels (*p* = 0.015; d = 1.608) and connective tissue cells (*p* = 0.017; d = 1.600). There was no significant difference in CTGF expression between connective tissue cells of control and CTS samples (Figure 6q).

### 3.7. Syndecan-1 Expression in Eccrine Sweat Glands

**Figure 7:**
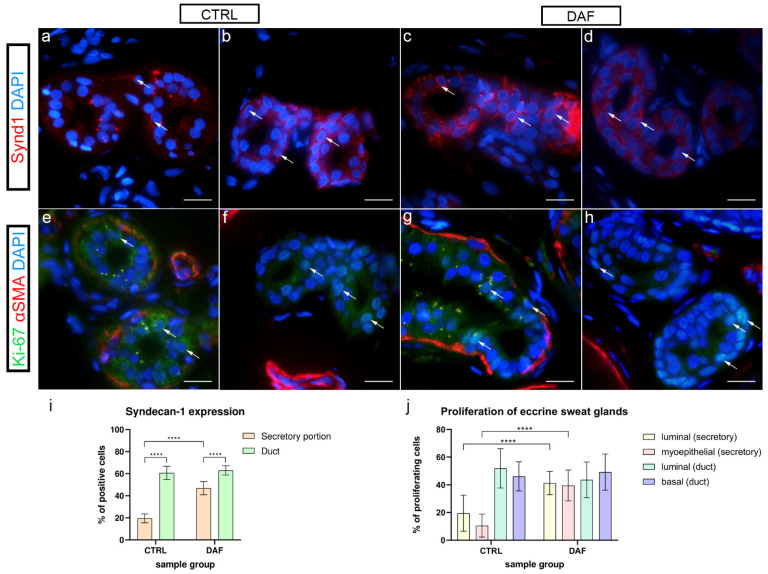
Expression of syndecan-1 and co-expression of Ki-67 and α-SMA in eccrine sweat glands of healthy controls and patients with DD. CTRL–normal skin of healthy individuals (**a**,**b**,**e**,**f**); DAF–clinically affected palmar fascia of DD patients (**c**,**d**,**g**,**h**). Syndecan-1 expression is seen in eccrine sweat gland cells (arrows) of secretory portions (**a**,**c**) and ducts (**b**,**d**). Nuclear Ki-67 expression is also seen in eccrine sweat gland cells (arrows) of secretory portions (**e**,**g**) and ducts (**f**,**h**); α-SMA staining shows myoepithelial cells of sweat gland secretory portions and sections through surrounding blood vessels (**e**–**h**). DAPI staining displays all cell nuclei (**a**–**h**). Immunofluorescence staining to syndecan-1 and DAPI, and double immunofluorescence staining to Ki-67, α-SMA, and DAPI, 1000× magnification, scale bar 50 µm. Statistically significant differences in syndecan-1 expression (**i**) and proliferation rate (**j**) are displayed by graphs. Error bars show standard deviation; **** *p* < 0.001.

In control samples, only a few cells of the secretory portions of eccrine sweat glands showed weak to moderate granular expression of syndecan-1 (Figure 7a), while in DAF samples, strong granular and membranous syndecan-1 expression characterized most of the cells (Figure 7b). The sweat gland ducts of control samples showed strong or moderate membranous expression of syndecan-1 in a majority of cells (Figure 7c), while ducts in DAF samples showed only moderate expression in cell membranes (Figure 7d). The secretory portions of control samples had an average of 19.54% ± 4.01% positive cells, which was significantly lower compared to both control sample ducts with 60.70% ± 6.02% positive cells (*p* < 0.0001; d = 8.046) and DAF sample secretory portions with 46.92% ± 5.99% positive cells (*p* < 0.0001; d = 5.369). The secretory portions of DAF samples had significantly fewer positive cells than DAF sample ducts with 63.00% ± 4.19% positive cells (*p* < 0.0001; d = 3.110). There was no statistically significant difference between the ducts of control and DAF samples (Figure 7i).

### 3.8. Proliferation of Eccrine Sweat Glands–Ki-67 and α-SMA Co-Expression

Here, α-SMA was applied to demonstrate differences between the secretory and ductal cells of sweat glands in control and DAF samples. In control samples, α-SMA staining demonstrated myoepithelial cells in secretory portions of eccrine sweat glands, while proliferating Ki-67 cells characterized several luminal and basal cells (Figure 7e). In ducts, myoepithelial cells were missing, while proliferating cells characterized their basal portions (Figure 7f). Compared to control samples, in DAF samples, proliferating Ki-67 cells were increased in secretory portions (Figure 7g), while ducts predominantly showed Ki-67 reactive cells in basal portions (Figure 7h). There was a statistically significant (*p* < 0.0001; d = 1.988) increase in Ki-67 positive luminal cells of secretory portions between control (19.53% ± 13.00%) and DAF samples (41.33% ± 8.46%). The difference in Ki-67 positive myoepithelial cells between control (10.53% ± 8.30%) and DAF samples (39.53% ± 11.18%) was also significant (*p* < 0.0001; d = 2.946). There was no statistically significant difference in Ki-67 expression between duct cells of control and DAF samples with around 40–50% of positive cells in both sample groups (Figure 7j).

### 3.9. FGFR1, FGFR2, CTGF, and TGF-β1 Expression in Eccrine Sweat Glands

#### 3.9.1. FGFR1 Expression

**Figure 8:**
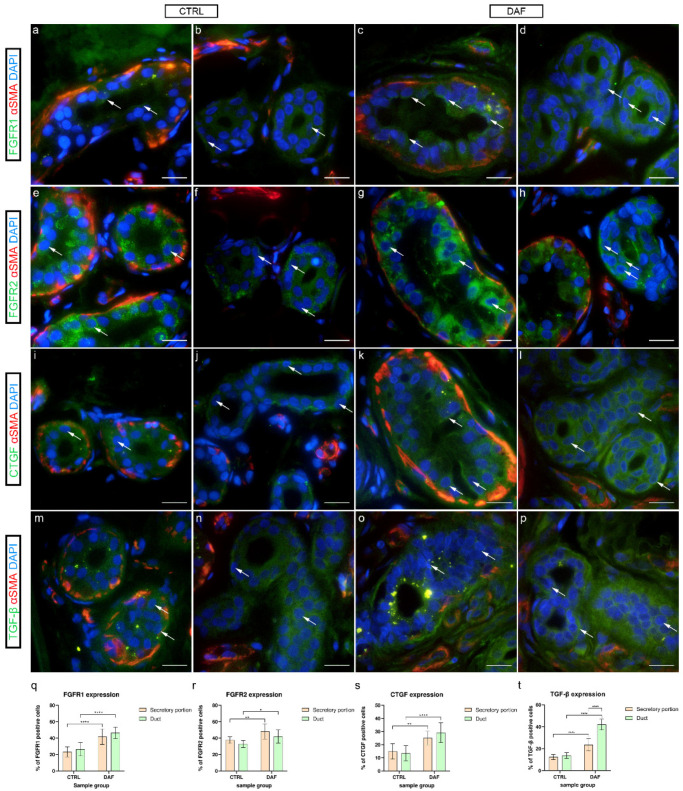
Co-expression of FGFR1/FGFR2/CTGF/TGF-β and α-SMA in eccrine sweat glands of healthy controls and patients with DD. CTRL–normal skin of healthy individuals (**a**,**b**,**e**,**f**,**i**,**j**,**m**,**n**); DAF–clinically affected palmar fascia of DD patients (**c**,**d**,**g**,**h**,**k**,**l,o**,**p**). FGFR1 expression is seen in sweat gland cells (arrows) of secretory portions (**a**,**c**) and ducts (**b**,**d**). FGFR2 expression is also seen in sweat gland cells (arrows) of secretory portions (**e**,**g**) and ducts (**f**,**h**). CTGF is expressed in sweat gland cells (arrows) of secretory portions (**i**,**k**) and ducts (**j**,**l**). TGF-β is also expressed in sweat gland cells (arrows) of secretory portions (**m**,**o**) and ducts (**n**,**p**); α-SMA staining shows myoepithelial cells of sweat gland secretory portions and sections through surrounding blood vessels (**a**–**p**). DAPI staining displays all cell nuclei (**a**–**p**). Double immunofluorescence staining to FGFR1/FGFR2/CTGF/TGF-β, α-SMA and DAPI, 1000× magnification, scale bar 50 µm. Graphs display statistically significant differences in FGFR1 (**q**), FGFR2 (**r**), CTGF (**s**), and TGF-β expression (**t**) between the analyzed samples. Error bars show standard deviation; * *p* < 0.05, ** *p* < 0.01, **** *p* < 0.001.

In control samples, granular cytoplasmatic FGFR1 expression was present in some secretory cells of sweat glands (Figure 8a), while it was weak in ducts (Figure 8b). In comparison, in DAF samples of sweat glands, moderate cytoplasmatic FGFR1 expression characterized several secretory cells (Figure 8c), while ducts showed moderate cytoplasmic expression (Figure 8d). FGFR1 expression was significantly higher (*p* < 0.0001; d = 2.319) in the DAF sample secretory portions (41.80% ± 9.48%) compared to the control sample secretory portions (23.30% ± 6.11%). It was also significantly higher (*p* < 0.0001; d = 2.633) in DAF sample ducts (46.50% ± 7.01%) compared to control sample ducts (26.40% ± 8.21%). There was no statistically significant difference between the secretory portions and ducts in either sample group (Figure 8q).

#### 3.9.2. FGFR2 Expression

In control samples of sweat glands, moderate to strong granular cytoplasmic FGFR2 expression characterized several secretory cells (Figure 8e), while FGFR2 was moderately expressed in the basal cells of ducts (Figure 8f). Compared to controls, in DAF samples, strong homogenous cytoplasmic expression of FGFR2 was present in most of the secretory (Figure 8g) and duct cells (Figure 8h). FGFR2 expression was significantly higher (*p* = 0.007; d = 1.483) in the DAF sample secretory portions (48.20% ± 9.28%) compared to the control sample secretory portions (37.60% ± 4.01%). It was also significantly higher (*p* = 0.024; d = 1.426) in DAF sample ducts (42.00% ± 7.94%) compared to control sample ducts (32.90% ± 4.28%). There was no statistically significant difference between the secretory portions and ducts in either sample group (Figure 8r).

#### 3.9.3. CTGF Expression

In control samples, weak cytoplasmic CTGF expression characterized secretory cells (Figure 8i), while ducts showed moderate to strong CTGF expression in the basal cytoplasm (Figure 8j). Comparatively, in DAF samples, moderate to strong cytoplasmic CTGF expression was seen both in the secretory cells (Figure 8k) and ducts (Figure 8l). CTGF expression was significantly lower (*p* = 0.004; d = 1.852) in the control sample secretory portions (14.90% ± 5.86%) compared to the DAF sample secretory portions (25.10% ± 5.13%). It was also significantly lower (*p* < 0.0001; d = 2.335) in control sample ducts (13.50% ± 5.74%) compared to DAF sample ducts (29.20% ± 7.58%). There was no statistically significant difference between the secretory portions and ducts in either sample group (Figure 8s).

#### 3.9.4. TGF-β1 Expression

In control samples, granular TGF-β1 expression was seen in secretory cells (Figure 8m), while moderate cytoplasmic expression characterized duct cells (Figure 8n). In contrast, the secretory portions of DAF samples showed stronger granular TGF-β1 expression (Figure 8o), while ducts displayed moderate to strong TGF-β1 expression in a majority of their cells (Figure 8p). TGF-β1 expression was significantly higher (*p* < 0.0001; d = 1.655) in the DAF sample secretory portions (23.50% ± 5.54%) compared to the control sample secretory portions (12.40% ± 2.27%). It was also significantly higher (*p* < 0.0001; d = 3.501) in DAF sample ducts (42.00% ± 4.94%) compared to control sample ducts (13.80% ± 2.62%). Secretory portions had a significantly lower signal area percentage compared to ducts in the DAF samples (*p* < 0.0001; d = 2.556), but not in control samples (Figure 8t).

## 4. Discussion

Our study focused on the comparison of the morphological characteristics, cell proliferation, and profibrotic signaling molecules in blood vessels and sweat glands between two diseases associated with tissue fibrosis—carpal tunnel syndrome and Dupuytren’s disease. We have analyzed healthy palmar fascia from CTS patients as control samples, as was previously described [37]. In contrast to most other studies, which have analyzed the subsynovial connective tissue of CTS patients [17,38,39], we have analyzed the flexor retinaculum to determine whether it is also affected by pathological changes. DD samples were separated into two groups, palmar fascia clinically unaffected by DD (DUF) and palmar fascia affected by DD (DAF), as previously described by Alfonso-Rodriguez et al. [40].

Our histological analysis of H&E samples revealed no significant differences between the flexor retinaculum and palmar fascia of CTS patients. However, DUF samples contained more dense connective tissue areas than the controls, while DAF samples demonstrated large areas of highly cellular connective tissue. Our study is in accordance with the study of Alfonso-Rodrigues et al. which also showed that DUF samples show initial morphological alterations [40], thus implying that DUF tissues are already histologically affected by DD. We have also described sweat glands between the fibrotic cords in DAF samples, which has been suggested to have a role in the pathogenesis of DD [18]. Similar to a previous study, Mallory trichrome staining of collagen revealed a gradual increase in the density of type I collagen packing from control through DUF to DAF samples [40].

In our study, we have not found significant differences in the proliferation of blood vessels or connective tissue cells of the flexor retinaculum of CTS patients compared to controls. However, other studies have proven increased fibroblast proliferation in CTS patients [41,42], as they have all analyzed subsynovial connective tissue instead of the flexor retinaculum. Compared to controls, in DUF samples proliferation was uniformly increased in endothelial, vascular smooth muscle, and surrounding connective tissue cells, while among the proliferating cells of DAF samples, the highest proliferation was observed in endothelial cells. In addition, some endothelial cells of DUF and DAF samples seemed morphologically changed, appearing more cuboid-like rather than squamous when observing their nuclear morphology. Other studies have also shown increased proliferation of vascular and connective tissue cells of DD samples [18,43]; however, they only analyzed DAF tissues, and the majority of proliferating cells were smooth muscle cells, not endothelial cells. We have additionally demonstrated the increased proliferation of eccrine sweat glands in DAF samples compared to healthy controls. However, increased proliferation in DAF samples compared to controls was found only in the secretory portions of the sweat glands and not the ducts, which is interesting given the fact that specifically the secretory portions were implicated in the pathogenesis of DD [18]. Considering that sweat glands are not present in the palmar fascia of control or DUF samples, we assume that the increased proliferation causes them to grow into the fascia of DAF samples.

In our study, we observed an increased expression of FGFR1 in both the wall of blood vessels and surrounding connective tissue cells of DUF and DAF samples, but not the flexor retinaculum of CTS patients, when compared to control samples. Increased expression of FGFR1 mRNA was previously demonstrated in DD tissues, but the distribution of the protein itself has not been previously described [44]. This is consistent with the role of FGFR1 in the stimulation of angiogenesis by mediating the proliferation of both endothelial and vascular smooth muscle cells [24,25]. The increased FGFR1 expression in the connective tissue cells has been previously explained by its upregulation mediated by TGF-β signaling, which leads to myofibroblast differentiation and increased extracellular matrix synthesis [45,46]. Myofibroblasts and TGF-β signaling seem to have a central role in the pathogenesis of DD [37,47]. We have also found increased FGFR1 expression in the sweat glands of DAF samples compared to controls, which may indicate that ERK signaling downstream of FGFRs may contribute to increased sweat gland proliferation [48].

FGFR2 expression in our study was mostly similar to FGFR1, with the exception of the flexor retinaculum of CTS patients having lower expression compared to controls. The increased FGFR2 expression in the blood vessel walls of DUF and DAF samples aligns with increased blood vessel proliferation, as FGFR2 contributes to angiogenesis by promoting cell migration [24]. Increased FGFR2 expression in the connective tissue of DD samples could contribute to DD-associated fibrosis as it has been shown that FGFR2 mediates fibrosis in both lungs and kidneys [49,50]. Interestingly, TGF-β has been shown to be able to both increase [51] and decrease FGFR2 expression [45]. TGF-β signaling was increased in fibrosis-affected tissues of both CTS and DD patients [17,37]; however, the flexor retinaculum adjacent to fibrotic CTS tissue did not display profibrotic changes, unlike the palmar fascia adjacent to fibrotic cords of DD patients. We, therefore, assume that fibrosis is mediated by different molecular mechanisms in CTS and DD; however, further studies of other profibrotic pathways are needed to confirm this. FGFR2 was also increased in the sweat glands of DUF samples compared to controls, probably also contributing to proliferation by the same mechanism as FGFR1 [48]. FGFR2 expression was previously described in the secretory portions of normal sweat glands, but not in the ducts [26]. We have demonstrated FGFR2 expression in the ducts of both control and DAF samples, albeit weaker than in secretory portions. This could be explained by the difference in the sensitivity of classic immunohistochemistry used in the referenced study and immunofluorescence used in our study [52].

The expression of CTGF is increased in the blood vessel walls and connective tissue cells of DUF and DAF samples compared to control samples, following the trend of FGFR1 and FGFR2. Slightly increased CTGF expression was also noted in the blood vessel walls of the flexor retinaculum of CTS patients, which could be explained with the same reasoning as the aforementioned decrease in FGFR2 expression since TGF-β has been also shown to stimulate CTGF expression [32]. We have also found increased CTGF expression in both secretory portions and ducts of DAF sample sweat glands compared to healthy controls. This partially corresponds to the findings of a previous study that exclusively described CTGF expression in the secretory gland portions [18]. The role of CTGF in tissue fibrosis and angiogenesis is well-known [31,33]; therefore it could contribute to the pathologic changes found in DD tissues.

The increased expression of all three analyzed factors (FGFR1, FGFR2, and CTGF) was already present in the seemingly unaffected palmar fascia of DD patients, even at higher levels than in their fibrotic cords. We assume that higher expression of profibrotic signaling molecules is required to prepare the tissue for initiation of ECM synthesis and formation of fibrotic cords, while maintenance of the fibrotic state can be accomplished at lower profibrotic signaling levels. This finding could explain the high relapse rate after surgical intervention, as only visibly fibrotic tissue is removed, while the clinically unaffected fascia, which has multiple profibrotic molecular changes, is spared [9,11]. Considering this, we suggest that a more extensive excision of palmar fascia be performed during routine surgical treatment of DD, not only removing fibrotic cords and nodules, but also the seemingly unremarkable and healthy-appearing fascia directly adjacent to them. We cannot comment on the usefulness of total fasciectomy in this regard, as we have only analyzed clinically unaffected fascia in close proximity to fibrotic cords, and additional studies analyzing fascia distal to visible fibrotic changes are needed to draw any meaningful conclusions. We also believe that these molecules could be targets for developing potential novel antifibrotic therapeutic strategies for preventing DD progression and reducing relapse rate.

The role of TGF-β1 in the pathogenesis of CTS and DD, as well as fibrosis in general, has been extensively analyzed [17,28,37]. However, TGF-β1 expression in DD-associated sweat glands has not been analyzed thus far. In our study, we have demonstrated TGF-β1 expression in both secretory portions and ducts of normal sweat glands and increased expression in both regions of DAF sweat glands. Increased expression of FGFR1, FGFR2, CTGF, and TGF-β1 in the ducts of the sweat glands indicates a potential role for them in the pathogenesis of DD, which is in contrast to a previous study which suggested that only secretory portions contribute to profibrotic signaling [18]. We propose that the increase in FGFR1, FGFR2, and CTGF expression in DD-associated sweat glands is related to their increased expression in blood vessels and connective tissue cells of the palmar fascia.

We have also analyzed the expression of syndecan-1 in sweat glands, as it has been shown that it can modify FGF signaling by associating with FGFRs and facilitating ligand binding [53]. Control sweat glands demonstrated only focal membranous expression in a few cells of their secretory portions, as had been previously described [54], while the secretory portions of DAF samples showed much stronger and widespread membranous syndecan-1 immunoreactivity. The ducts of both analyzed groups displayed strong membranous staining and increased syndecan-1 expression compared to their respective secretory portions. The increased syndecan-1 expression in DD-associated sweat glands probably contributes to their FGFR-mediated signaling [53].

## 5. Conclusions

We have found increased proliferation and FGFR1, FGFR2, and CTGF expression in the blood vessel walls and connective tissue cells of DUF and DAF samples, indicating that molecular changes associated with DD are present even in the seemingly unaffected palmar fascia. Only minor changes in FGFR2 and CTGF expression were found in the flexor retinaculum of CTS patients, suggesting it is not the primarily affected tissue in CTS. All of the aforementioned factors, as well as TGF-β and syndecan-1, were also increased in sweat glands associated with DD cords, implicating their possible role in the pathogenesis of DD.

## Figures and Tables

**Table 1:** Primary and secondary antibodies used in the study.

	Antibodies	Host	Code No.	Dilution	Source
Primary	Anti-Ki-67	Rabbit	AB9260	1:300	Sigma-Aldrich, Inc., St. Louis, MO, USA
	Flg (Anti-FGFR1)	Rabbit	sc-121	1:25	Santa Cruz Biotechnology, Dallas, TX, USA
	Bek (Anti-FGFR2)	Rabbit	sc-122	1:50	Santa Cruz Biotechnology, Dallas, TX, USA
	Anti-CTGF	Goat	sc-14939	1:50	Santa Cruz Biotechnology, Dallas, TX, USA
	Anti-TGF-β1	Rabbit	ab215715	1:100	Abcam, Cambridge, UK
	Anti-Syndecan-1	Mouse	ab34164	1:75	Abcam, Cambridge, UK
	Anti-α-SMA	Mouse	M0851	1:300	DAKO, Glostrup, Denmark
Secondary	Alexa Fluor^®^ 488 Anti-Rabbit lgG	Donkey	711-545-152	1:400	Jackson Immuno Research Laboratories, Inc., Baltimore, PA, USA
	Alexa Fluor^®^ 488 Anti-Goat lgG	Donkey	705-545-003	1:400	Jackson Immuno Research Laboratories, Inc., Baltimore, PA, USA
	Rhodamine Red™-X Anti-Mouse IgG	Donkey	715-295-151	1:400	Jackson Immuno Research Laboratories, Inc., Baltimore, PA, USA

## Data Availability

The data presented in this study are available from the corresponding author upon reasonable request.
